# Accuracy of an Accelerated, Culture-Based Assay for Detection of Group B *Streptococcus*

**DOI:** 10.1155/2013/367935

**Published:** 2013-02-19

**Authors:** Jonathan P. Faro, Karen Bishop, Gerald Riddle, Mildred M. Ramirez, Allan R. Katz, Mark A. Turrentine, Sebastian Faro

**Affiliations:** ^1^Department of Obstetrics, Gynecology and Reproductive Sciences, The University of Texas Health Science Center, 6411 N Fannin Street, Houston, TX 77030, USA; ^2^Department of Obstetrics and Gynecology, Baylor College of Medicine, Houston, TX 77030, USA; ^3^Department of Obstetrics and Gynecology, Kelsey-Seybold Clinic, Houston, TX 77025, USA

## Abstract

*Objective*. To determine the validity of a novel Group B *Streptococcus* (GBS) diagnostic assay for the detection of GBS in antepartum patients. *Study Design*. Women were screened for GBS colonization at 35 to 37 weeks of gestation. Three vaginal-rectal swabs were collected per patient; two were processed by traditional culture (commercial laboratory versus in-house culture), and the third was processed by an immunoblot-based test, in which a sample is placed over an antibody-coated nitrocellulose membrane, and after a six-hour culture, bound GBS is detected with a secondary antibody. *Results*. 356 patients were evaluated. Commercial processing revealed a GBS prevalence rate of 85/356 (23.6%). In-house culture provided a prevalence rate of 105/356 (29.5%). When the accelerated GBS test result was compared to the in-house GBS culture, it demonstrated a sensitivity of 97.1% and a specificity of 88.4%. Interobserver reliability for the novel GBS test was 88.2%. *Conclusions*. The accelerated GBS test provides a high level of validity for the detection of GBS colonization in antepartum patients within 6.5 hours and demonstrates a substantial agreement between observers.

## 1. Introduction

Group B *Streptococcus* (GBS) has been shown to cause significant neonatal morbidity, and current national guidelines recommend antenatal screening be performed on all pregnant women between 35 to 37 weeks gestation [1]. Current culture methods for GBS require 48 hours for identification, and if antibiotic sensitivity is necessary, an additional 24 hours may be needed [2]. Culture of GBS is routinely performed on agar supplemented with sheep's blood, and colonies are detected by the breakdown of red blood cells, producing a characteristic zone of hemolysis, termed beta hemolysis [3]. The presence of GBS may be confirmed by performing Gram stain, CAMP testing, or typing via an agglutinin reaction on selected beta-hemolytic colonies [1]. This is often labor intensive, and in cases in which the patient's sample contains many different species, beta hemolysis may be missed [4–6]. There is the risk that samples may be overgrown with competing organisms, such as *Proteus* or *Enterococcus*, and false negatives are not uncommon. When hemolysis is identified, follow-up testing is often required to prevent a false positive from being reported [7]. While culture continues to be regarded as the gold standard, it is labor intensive and is not without shortcomings.

Several options are available which attempt to address the deficiencies inherent with traditional culture techniques. The most notable of these includes the use of specialized media containing a chromogen, allowing the reader to be relatively certain of the identification [6]. Another recommendation is that an 18–24-hour enrichment step be used prior to culture [1, 8]. While PCR continues to show promise for use in intrapartum testing, current CDC guidelines allow for PCR to be performed in an antepartum setting, but the guidelines caution against the use of PCR alone when susceptibility testing is required and recommend that in situations when anaphylaxis is a concern in a penicillin-allergic patient, culture be performed as well [1].

We have recently reported on the development of an antibody-based diagnostic test which allows for GBS present in a patient vaginal-rectal specimen to bind selectively to an antibody-coated nitrocellulose membrane [9, 10]. After the organism is isolated and competing microbes are removed, a short culture step is employed. Following this, any bound GBS is detected by the addition of a labeled antibody directed against GBS. An important additional feature of this test is that it allows for the simultaneous identification of GBS and determination of its antimicrobial susceptibility [11]. Our objective was to determine the validity of this GBS test as it relates to the identification of GBS in routine screening of antepartum patients. 

## 2. Material and Methods

 The clinical protocol was developed along with STARD guidelines [12]. Institutional review board approval was obtained from the University of Texas Health Science Center at Houston, and the study was registered at http://ClinicalTrials.gov/ (NCT01445717). Patients were recruited from the obstetrical clinics at the University of Texas Health Science Center, Houston. Three vaginal-rectal swabs were collected in Amies Transport tubes during the patients' 35 to 37 weeks of antenatal visit. Demographic data was recorded, including the patient's age and ethnicity. The first swab was sent for routine GBS culture processing at a commercial laboratory (Quest Diagnostics, Madison, NJ) and was utilized for patient management. The second and third swabs were transferred to our laboratory at the University of Texas Medical School. These two swabs were processed by either GBS culture or by the GBS accelerated test as described below. 

For GBS culture, the second swab was inoculated into LIM broth and incubated overnight. A sample was then used to inoculate a CNA plate (colistin and nalidixic acid, BD Biosciences, Cockeysville, MD). A primary, nonenriched culture was also performed on a GBS Detect plate (Hardy Diagnostics, Santa Maria, CA). These were incubated at 37°C overnight, and plates showing characteristic colony morphology, hemolysis, and catalase reaction were tested further. GBS was confirmed by the use of the PathoDx test (Remel, Lenexa, KS). The third swab was processed by the antibody-based accelerated test (QuickTest, Nanologix, Inc. Hubard, OH) as previously described [9, 10]. Interpretation of the GBS accelerated test was performed in a blinded fashion without knowledge of the other two culture results. A second independent reader interpreted the GBS accelerated test, without knowledge of culture results or the initial GBS test result for the determination of interobserver reliability of the GBS accelerated test assay. 

 Sample size was based on the proportion of concordant responses to a reference sample (GBS culture). The sample size was calculated to have an 80% power (alpha = 0.05) to determine at least a 10% difference in sensitivity between the GBS accelerated test and GBS culture. Assuming that GBS culture is the “gold standard” and detects 100% of GBS colonization, if the accelerated GBS test is 90% sensitive for GBS, approximately 88 women colonized with GBS would have to be screened. If one assumes a prevalence of GBS during pregnancy of 25%, then 352 women would need to be tested. 

The prevalence of GBS was determined by test results obtained by the commercial laboratory GBS culture, in-house GBS culture, and the accelerated GBS test from the initial interpretation. The Centers for Disease Control and Prevention guidelines recommend the use of an enrichment phase and specialized culture media in the detection of GBS [1]. Since this technique has been shown to be superior to routine culture, analysis of the accelerated GBS test was compared to the results obtained by our in-house GBS culture technique [1]. For the accelerated GBS test, a true-positive result was defined as positive by the accelerated test and positive by the in-house GBS culture. A false-positive result was defined as positive by the accelerated test and negative by the in-house GBS culture. A true-negative result was defined as negative by the accelerated test and negative by the in-house GBS culture. A false-negative result was defined as negative by the accelerated test and positive by the in-house GBS culture. The sensitivity, specificity, positive predictive value, and negative predictive value with 95th percentile confidence intervals (95th% CI) were determined. The inter-observer reliability was determined for the results obtained by the two independent readers of the accelerated GBS test with Kappa scoring. 

## 3. Results

Between March 2011 and May 2012, 356 women were enrolled. Informed consent was obtained, and all women had three vaginal-rectal swabs performed and processed. The mean gestational age at study enrollment was 35.6 weeks of gestation. The mean age of women was 26.8 ± 0.6 years. The self-designated ethnic distribution of patients was African American 178 (50%), Hispanic 85 (23.9%), Caucasian 79 (22.2%), Asian 12 (3.4%), and “Other” 2 (0.6%). The mean age for those screening positive for GBS was similar to that of patients who screened negative (27.2 versus 26.7 years, resp., *P* = 0.45). The prevalence of GBS colonization as determined by the in-house culture stratified by ethnic group was African American 72/178 (40.4%), Hispanic 16/85 (18.8%), Caucasian 14/79 (17.7%), Asian 2/12 (16.7%), and “Other” 1/2 (50%).

Utilizing the in-house GBS culture as the reference standard, we compared the sensitivity, specificity, positive predictive value, and negative predictive value with 95th percentile confidence intervals for the commercial laboratory GBS culture and the initial accelerated GBS test result (Table 1). The time from the initiation of the GBS accelerated test until a result was available was 6.5 hours. The estimation of inter-observer reliability for the accelerated GBS test demonstrated that the number of observed agreements between the two blinded-independent readers was 314 (88.2%), which was significantly higher than the number of results expected to be in agreement, 178 (50%). The Kappa score estimate for the inter-observer reliability showed substantial agreement at a value of 0.76 (95th% CI 0.70–0.83).

## 4. Discussion

In this study of diagnostic accuracy, the accelerated GBS test demonstrated a high level of sensitivity and specificity for the detection of antepartum GBS within 6.5 hours and a substantial agreement between observers. Identification of a microbe by culture has traditionally involved plating out on solid-phase agar and then overnight incubation at 37°C. Selective media have been used to limit the growth of competing organisms and has been successfully utilized to aid in the identification of GBS [13]. Additional steps that have assisted the laboratory clinician in including enrichment in liquid media, as well as the use of chromogenic agar [1, 2, 5, 7, 8]. All of these steps have been proven to increase both the sensitivity and specificity when identifying GBS from a patient's vaginal-rectal specimen and have been approved for use by the Centers for Disease Control and Prevention [1]. The current assay is a culture-based method for detection of bacteria using an antibody labeled with a chromogen and allow bacteria to be grown and detected in a much shorter period of time than the traditional agar-based methods. This newly developed GBS test has demonstrated that a purified culture of GBS may be detected at various time points [9]. We have shown that greater dilutions of GBS may be detected with greater periods of incubation, with a clinically relevant concentration of GBS (10^4^) being detectable as early as 4 hours [9]. We previously determined that the six-hour incubation period utilized in this study increases the sensitivity of this test [9]. Other advantages are that no additional time is required for enrichment of samples and the sensitivity of the GBS accelerated test is comparable to enriched culture for detecting GBS. Further, antimicrobial susceptibility testing for penicillin-allergic women is possible [11].

Yet, our study has some limitations. The incubation step involved in this assay limits the testing of intrapartum women. Test results would potentially not be available in time to provide adequate intrapartum antibiotic prophylaxis for neonatal exposure to GBS. Further, since the assay result is presented as a visual dot on a nitrocellulose membrane, there is a potential risk of poor inter-observer reliability. However, our blinded assessment of this showed substantial agreement between observers [14]. One potential solution may be the conversion of the current test to an enzyme-linked immunosorbent assay so that an optical density reading is given. Preliminary results, utilizing such a method, have reduced result availability time to within 60 minutes [15]. Similar limitations have been noted with nucleic acid amplification techniques (i.e., polymerase chain reaction) in diagnosing colonization of GBS [16–19]. While initial reports found polymerase chain reaction to be less promising than anticipated; more recent studies have demonstrated good performance allowing for results in 45 minutes [20]. Yet, this newer polymerase chain reaction test still resulted in a large number (13%) of indeterminate samples which required reprocessing. 

In our evaluation, the accelerated GBS test was found to perform well for the detection of GBS in antepartum patients, was easy to use, and had the unique potential for applying antibiotic resistance testing during the culture step. These characteristics may allow for the implementation of this test across a wide range of clinical scenarios in geographical settings where access to specialized equipment is limited. By modifying the antibodies used during this test, one may substitute specificity for GBS with other organisms, such as *Escherichia coli*, *Neisseria gonorrhoeae*, or *Enterococcus*. The potential exists for the simultaneous identification of a microbe and determination of its resistance profile, so that a clinician may be armed with both of the identification of the agent causing infection and the appropriate antibiotic needed to provide optimal treatment. This not only has the potential of saving time and resources, but will also allow for improved clinical outcomes and reduce the burden of microbial resistance which may develop from empirical prophylaxis.

## 5. Conclusions

Antepartum GBS colonization was identified in 6.5 hours with a novel immunoblot-based test using an antibody-coated nitrocellulose membrane with a high level of validity and demonstrating substantial agreement between observers.

## Figures and Tables

**Table 1:** Antepartum validity of the accelerated GBS test compared to GBS culture.

GBS test (*n* = 356)	Prevalence	Sensitivity	Specificity	PPV	NPV
In-house culture	105 (29.5%)	reference			
Commercial culture	84 (23.6%)	73.3 (63.7–81.3)	97.2 (94.1–98.8)	91.7 (83.0–96.3)	89.7 (85.3–92.9)
Accelerated GBS test	131 (36.8%)	97.1 (91.3–99.3)	88.4 (83.7–92.0)	77.9 (69.6–84.4)	98.7 (95.8–99.7)

Numbers are *n* (%). Data are % (95th percentile confidence interval).

PPV: positive predictive value; NPV: negative predictive value.
